# Rhinovirus Genome Evolution during Experimental Human Infection

**DOI:** 10.1371/journal.pone.0010588

**Published:** 2010-05-11

**Authors:** Samuel Cordey, Thomas Junier, Daniel Gerlach, Francesca Gobbini, Laurent Farinelli, Evgeny M. Zdobnov, Birgit Winther, Caroline Tapparel, Laurent Kaiser

**Affiliations:** 1 Laboratory of Virology, Division of Infectious Diseases and Division of Laboratory Medicine, University of Geneva Hospitals, Geneva, Switzerland; 2 Medical School, University of Geneva, Geneva, Switzerland; 3 Department of Genetic Medicine and Development, University of Geneva Medical School, Geneva, Switzerland; 4 Swiss Institute of Bioinformatics, Geneva, Switzerland; 5 Fasteris SA, Plan-les-Ouates, Switzerland; 6 Division of General Pediatrics, Department of Pediatrics, University of Virginia, Charlottesville, Virginia, United States of America; Institute of Infectious Disease and Molecular Medicine, South Africa

## Abstract

Human rhinoviruses (HRVs) evolve rapidly due in part to their error-prone RNA polymerase. Knowledge of the diversity of HRV populations emerging during the course of a natural infection is essential and represents a basis for the design of future potential vaccines and antiviral drugs. To evaluate HRV evolution in humans, nasal wash samples were collected daily for five days from 15 immunocompetent volunteers experimentally infected with a reference stock of HRV-39. In parallel, HeLa-OH cells were inoculated to compare HRV evolution in vitro. Nasal wash in vivo assessed by real-time PCR showed a viral load that peaked at 48–72 h. Ultra-deep sequencing was used to compare the low-frequency mutation populations present in the HRV-39 inoculum in two human subjects and one HeLa-OH supernatant collected 5 days post-infection. The analysis revealed hypervariable mutation locations in VP2, VP3, VP1, 2C and 3C genes and conserved regions in VP4, 2A, 2B, 3A, 3B and 3D genes. These results were confirmed by classical sequencing of additional samples, both from inoculated volunteers and independent cell infections, and suggest that HRV inter-host transmission is not associated with a strong bottleneck effect. A specific analysis of the VP1 capsid gene of 15 human cases confirmed the high mutation incidence in this capsid region, but not in the antiviral drug-binding pocket. We could also estimate a mutation frequency in vivo of 3.4×10^−4^ mutations/nucleotides and 3.1×10^−4^ over the entire ORF and VP1 gene, respectively. In vivo, HRV generate new variants rapidly during the course of an acute infection due to mutations that accumulate in hot spot regions located at the capsid level, as well as in 2C and 3C genes.

## Introduction

Human rhinoviruses (HRV) are the most frequent cause of respiratory infection in humans [1]. These viruses belong to the *Picornaviridae*, one of the oldest and most diversified human virus family, characterized by a non-enveloped, single positive-stranded RNA genome. Although rhinovirus replication is often restricted to the upper respiratory tract leading to self-limited illnesses of short duration, such as the common cold, HRV can also invade the lower respiratory tract and lead to more serious infections [2], [3].

Similar to many other RNA viruses, the error-prone rhinoviral polymerase can accumulate a large number of nucleotide mutations over a very short period of time, a feature that favors viral adaptation. The error rate of picornavirus RNA polymerases has been estimated to range between 10^−3^ and 10^−4^ errors/nucleotide/cycle of replication [4], [5]. This variability is a driving force for virus evolution and results in a large genetic and phenotypic diversity illustrated by the very high number of different HRV serotypes identified to date (http://www.picornaviridae.com/enterovirus/enterovirus.htm). As for other RNA viruses, the adaptive immune-mediated positive selection, which targets the capsid region for rhinoviruses, is probably one of the main HRV evolutionary forces at both the intra- and inter-host levels. The in vivo selection of resistant variants during exposure to anti-VP1 agents confirms the virus ability to rapidly mutate the capsid protein while still conserving replicative fitness [6]–[8]. The different environmental conditions of the upper and the lower respiratory tracts could also impact on internal genes. These observations are consistent with the full-length genome data of all HRV serotypes that show the capsid genes VP2, VP3 and VP1 as the least conserved [9].

Rhinovirus species and their respective serotypes have thus emerged due to the ability to accumulate a large number of mutations along the whole genome while preserving both replicative capacity and transmissibility. Opportunities for these mutational events likely occur within the context of each human infection that is usually limited to only a few days. Therefore, it is important to study the patterns and kinetics of viral genome evolution during the course of HRV infection in individuals, although this does not take into account recombination events that are also used by rhinoviruses to generate new species [10]. In addition, it is likely that the respiratory mucosal surface is exposed to a cloud of different variants of a quasispecies after deposition of infectious droplets. This raises the question of whether rhinovirus infection in humans results from the selection of a given clone among the quasispecies (bottleneck effect) or from concomitant infection by several different variants that are part of the transmitted quasispecies population. Rhinovirus-positive clinical samples collected in humans with naturally-acquired respiratory disease are not suitable for such a study as the time elapsed since the beginning of the infection remains unknown. For this reason, we took advantage of samples from experimentally inoculated adult volunteers to assess the genome evolution over a 5-day course of infection, which is likely to represent the peak window period of transmissibility. In parallel, we infected HeLa-OH cells with the same HRV-39 inoculum to compare in vitro and in vivo adaptation.

The classical Sanger sequencing method usually reliably detects viral variants present at a frequency of at least 20% within a heterogeneous virus population [11], [12]. However, the development of ultra-deep sequencing technologies, such as pyrophosphate-based sequencing (pyrosequencing) or reversible chain-terminator extension, now allows efficient detection of viral variants present in only 1–2% of the population [13]. In the present investigation, we used both methods and compared results.

The aim of our study was to describe the HRV-39 genome evolution over a 5-day period in its natural host and to analyze the kinetics of minority mutations (i.e. low-frequency mutations present between 2–50% within the viral population) in vivo and in vitro following inoculation with a quasispecies cloud of viral variants. This allowed us to point out regions along the HRV-39 open reading frame (ORF) that were enriched for mutations or conserved, and to determine whether the HRV genome evolution after 5 days of infection shares similarities with the general long-term HRV evolution history. Collectively, these results should contribute to evaluate the ability of HRV to generate new variants, as well as their ability to escape antivirals and vaccines.

## Results

### Viral load and HRV-39 kinetics infection in inoculated subjects

**Figure 1:**
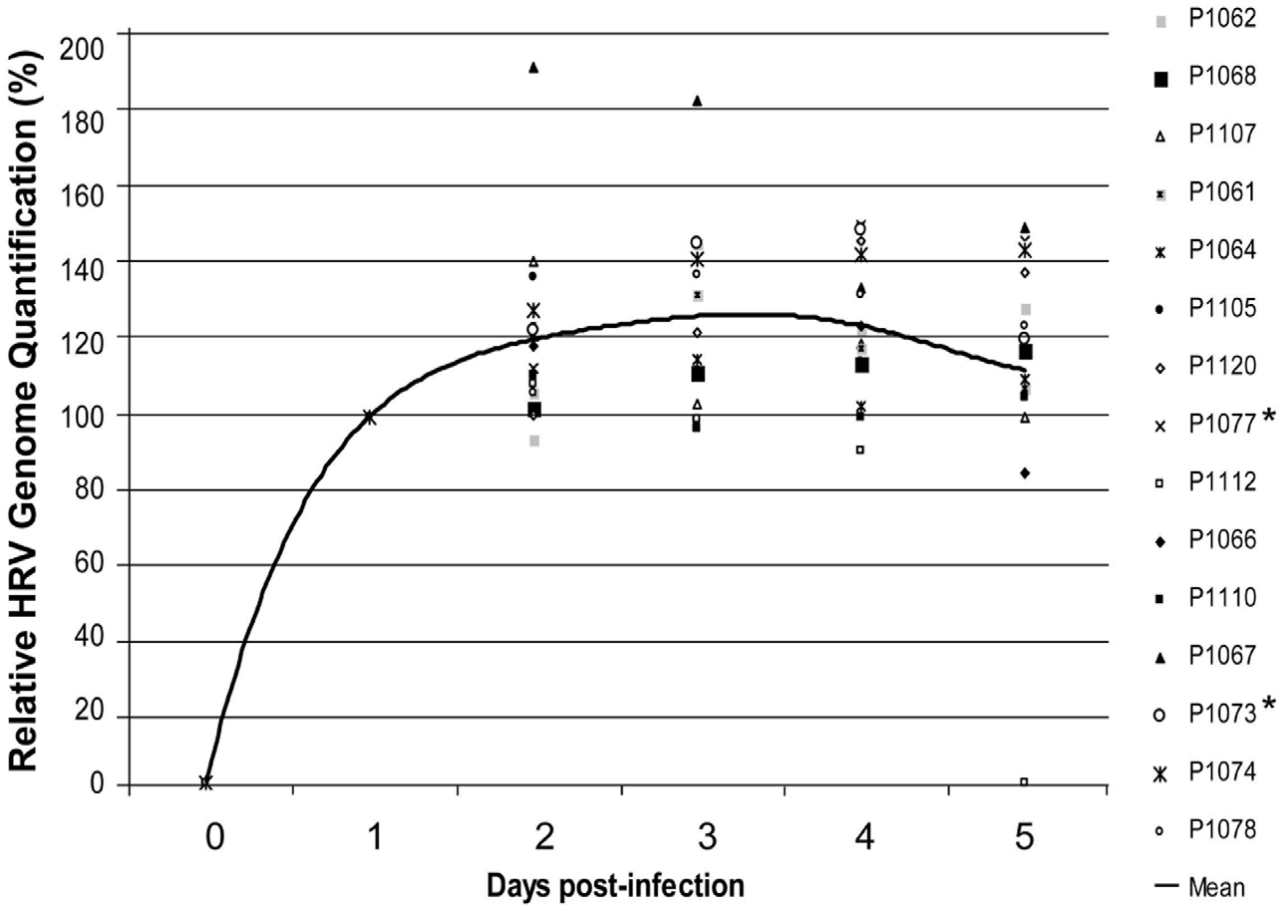
Kinetics of HRV-39 infection in inoculated patients. The course of infection is shown from days 0 to 5 for each patient (P). The relative HRV RNA values are expressed as 1/C_T_ and converted into % with 100% being arbitrarily set for each patient at day 1. The mean value is represented by the black line. Subjects analyzed further by ultra-deep sequencing are marked with an asterisk.

Nineteen human volunteers were infected with a standardized quantified inoculum (∼1000 50% tissue culture infective dose/mL) of an HRV-39 viral stock. We then collected nasal wash (NW) samples from days 0 to 5 to assess the kinetics of the HRV-39 viral load by real-time PCR. Four of 19 subjects were found to be rhinovirus RNA- positive the day prior to inoculation, either from a recent or ongoing infection, and were excluded from this analysis. As expected, real-time PCR performed on NW samples were positive for all but one subject at day 5. Although differences in HRV-39 viral loads were observed between individuals, viral titers peaked consistently between days 2 and 3 before starting to decline (Fig. 1). These results confirm previous observations with HRV-16 inoculation that relate a similar peak 48 h post-infection correlating with the peak of symptom scores [14].

### Mutation analysis along the whole ORF by ultra-deep and classical sequencing methods

#### Generation of new mutations in the course of infection and adaptation to a specific host

The dynamics of HRV-39 minority mutation evolution were analyzed by ultra-deep sequencing of three samples collected at day 5 post-infection (two clinical, one in vitro) by comparing the entire ORF of the initial HRV-39 inoculum with the sequence obtained in two human subjects (P1073 and P1077) and in HeLa-OH cells (HeLa-A). To exclude mutation introductions linked to RT or DNA polymerase errors, each RNA extracted from human NW or HeLa-OH cell samples was reverse transcribed in duplicate and amplified by PCR in quadruplicate. The presence of single nucleotide mutations identified by the ultra-deep sequencing approach was considered only if statistically reproducible in each of our replicates (see Materials and Methods).

**Figure 2:**
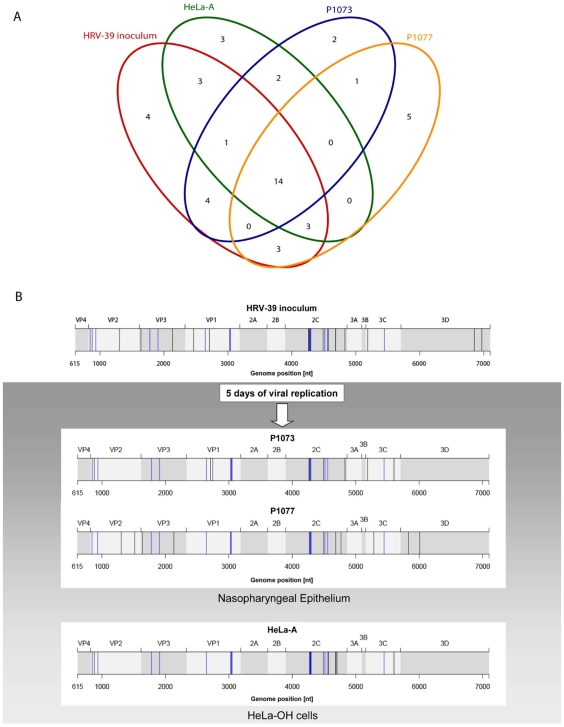
Representation of all specific or common minority mutations to be found in the same loci for the four samples analysed by ultra-deep sequencing. (A) Venn plot showing the minority mutations present initially in the HRV-39 inoculum and those present in HeLa-A, P1073 and P1077 after 5 days of infection. (B) Minority mutations present in the initial inoculum and in subjects P1073 and P1077 after 5 days of viral replication in the nasopharyngeal epithelium are represented by black and blue at their respective positions along the HRV-39 ORF. Blue bars represent the 14 minority mutations present in the Venn plot in the four viral populations.

A Venn diagram provides an overview of the repartition of minority mutations in the four different viral populations analysed and shows individual overlaps (Fig. 2A). The total number of minority mutations detected in the four viral populations was 45. The number of minority mutations detected in the original inoculum was 32, thus revealing that 13 additional minority mutations appeared in the three populations studied after 5 days of viral replication. Most minority mutations present in the inoculum were detected in at least one of the two subjects studied (25/32) with 14 kept within the two viral populations (Fig. 2A, B), thus suggesting that most initial variants had passed in these infected volunteers. Similar results were observed in HeLa-OH cells (21/32 minority mutations conserved, Fig. 2A, B). Viral populations present in NW samples of subjects P1073 and P1077 and the cell supernatant were composed of 24, 26 and 26 minority mutations, respectively, with 21%, 23% and 19% representing new minority mutations having emerged during the 5-day infection period.

During the course of infection, four minority mutations present in the inoculum were counter-selected (one in VP1, one in 2C NTPase A site [15], two in 3D) and two, five and three appeared specifically in patients P1073 (one in VP1 NIm-IB domain [16], one in 2C NTPase A site), P1077 (one in VP2, one in 2C and three in 3D) and in HeLa-A (two in 2C NTPase A site, one in 2C zinc finger motif [17]), respectively, and were present at similar frequencies compared to all minority mutations. Finally, two minority mutations were present both in HeLa-A and P1073 (one in VP1, one in 3C) and one in both P1073 and P1077 (located in 2C). Overall, we did not observe a significant difference in terms of the number of new mutations appearing in human and HeLa-OH cells.

#### Distribution of mutations along the ORF

**Figure 3:**
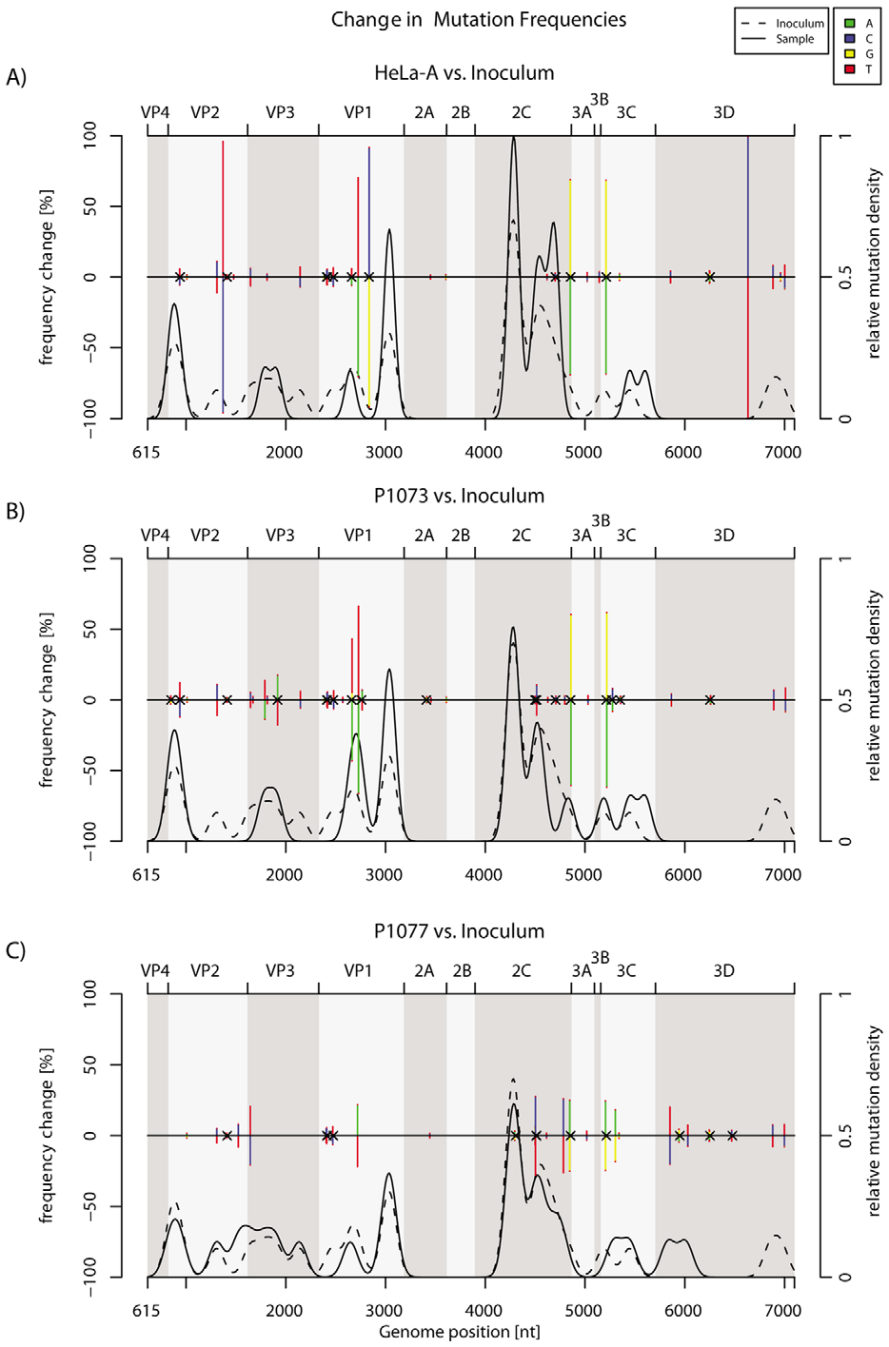
Change in mutation frequencies (minority and majority mutations, colored bars) and minority mutation densities (curves). Colored bars represent the difference in proportions of each nucleotide between the inoculum and the final (5 days' post-infection) sample in HeLa (A), subjects P1073 (B) and P1077 (C). As each gain in proportion by one nucleotide must be a loss by another, the changes sum to zero. Crosses indicate non-synonymous mutations. Curves indicate minority mutation densities (estimated by a Gaussian kernel function), including mutations whose nucleotide proportions did not change between the inoculum and the final sample.

We estimated the initial and final densities of minority mutations along the ORF with kernel functions using the ultra-deep sequencing data and were able to identify regions with a higher density of mutations (Fig. 3). The distribution and pattern of these spots along the ORF were similar between the inoculum and the viral populations analyzed after 5 days of replication in humans or cells. Most minority mutations are located within VP2, the second half of VP1, and the 2C genes (see filled and dashed curves, Fig. 3A, B, C). Interestingly, no minority mutations were detected within the VP1 drug-binding pocket [6], thus suggesting a relative stability of this structure, at least in the absence of specific pressure. This analysis also identified the presence of regions that were highly conserved: in VP4, from the start of 2A to the end of 2B; in 3A, 3B genes; and finally in 3D (the polymerase gene).

Comparison of the P1073, P1077 or HeLa-A sample sequences with that of the initial HRV-39 inoculum was then performed to determine both the presence of the new majority species (defined as a frequency change of over 50%) and the amplitude of all mutation frequency changes in the population after 5 days' infection (Fig. 3A, B, C, colored bars). This analysis shows the presence of three majority nucleotide changes along P1073 ORF, none for P1077, and six for HeLa-A. Both the amplitude and the number of mutation (minority and majority mutations) frequency changes were similar between P1073, P1077 and HeLa-A and mainly located within regions previously identified as being enriched of mutations. Interestingly, all 14 minority mutations present in the four viral populations in Fig. 2 are mainly located within these highly variable regions (VP1 [4/14], VP2 [2/14], 2C [5/14], VP3 [2/14] or 3C [1/14]), but not in regions depleted of mutations identified above.

#### Majority species after five days of infection

**Figure 4:**
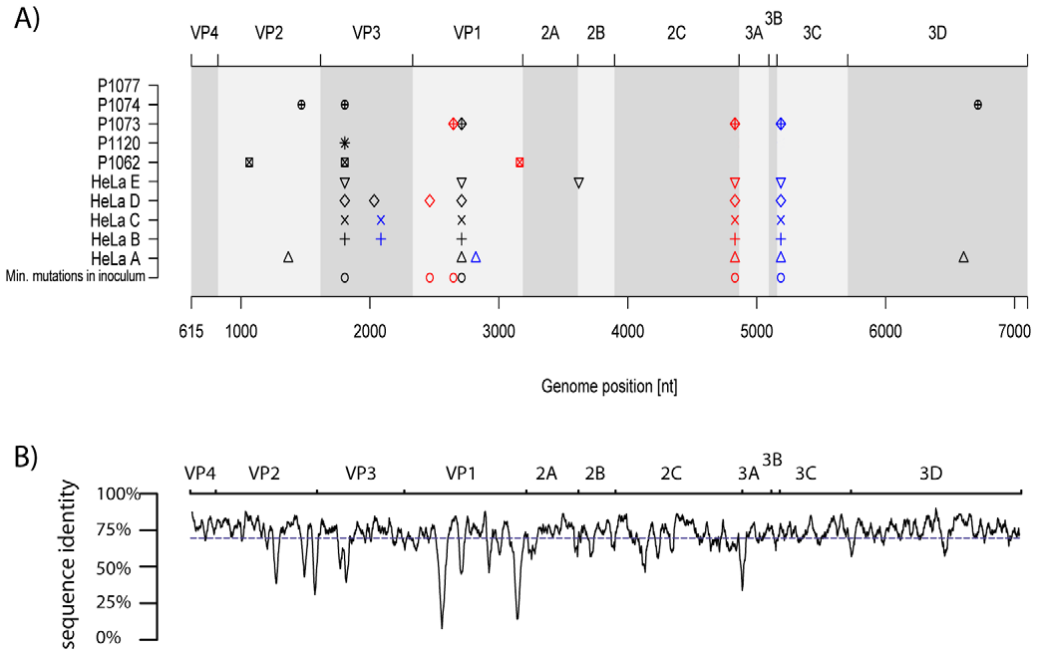
Comparison of ORF consensus sequences in HeLa-OH and human volunteers. (A) Five HeLa-OH (A to E) and samples from five human volunteers (P1062, P1120, P1073, P1074 and P1077) collected 5 days' post-infection were analysed by classical sequencing method. All mutants present after 5 days of infection, as well as those already present as minority mutations in the initial HRV-39 inoculum, are shown at their respective genome position. Synonymous, non-synonymous and non-conservative mutations are represented by black, and blue and red symbols, respectively. (B) The conservation plot was calculated based on an alignment of 99 rhinovirus serotypes as previously published [9]. The average sequence identity for the ORF was 69.9% (blue dashed line).

We performed also Sanger sequencing in parallel on the entire ORF of the specimens analyzed above, as well on NW samples from three additional patients (P1062, P1120 and P1074) and cell supernatants from four additional experiments (HeLa-B to E). Again, consensus sequences after 5 days of viral replication were compared to the inoculum sequence (Fig. 4A). This analysis confirmed the ultra-deep sequencing as all the nucleotide changes identified previously for P1073, P1077 and HeLa-A samples are strictly those identified by the Sanger sequencing. A total number of four non-synonymous (two non-conservative, present both in P1073 at the full-length positions 2647 in VP1 and 4831 in 2C) and seven synonymous mutations were found in samples from human subjects. Similar to the ultra-deep analysis, most mutations are located within the viral capsid genes VP1 (3/11), VP2 (2/11) and VP3 (3/11), while VP4 (0/11), 2A (0/11), 2B (0/11), 3A (0/11), 3B (0/11) and 3D (1/11) regions were confirmed to represent relatively conserved regions with few or no mutations identified.

Fourteen non-synonymous (six non-conservative: one present in HeLa-D at the HRV-39 full-length position 2464 in VP1, and one present in the five HeLa-OH experiments at position 4831 in 2C) and 13 synonymous mutations were found in cells. Again, most mutations (15/27) are located within the viral capsid genes VP2, VP3 and VP1, while no mutations were present in VP4, 2A, 3A, 3B, and only one in 2B and 3D. None of the mutations observed both in vivo and in vitro is located in HRV domains with known functions [15]–[21].

Finally, taking into account the number of patients or cells analysed and the length of the ORF, we estimate that the occurrence of major mutations after 5 days of HRV-39 infection in human subjects is of 3.4×10^−4^ mutations/nucleotides (total of 11 mutations/[5 subjects ×6443 nucleotides analysed]) versus 8.4×10^−4^ in cells (total of 27 mutations/[5 HeLa-OH assays ×6443 nucleotides analyzed]).

### In vivo and in vitro VP1 sequence analysis

The capsid protein VP1 is responsible for receptor binding and is also a target for certain antivirals. In addition, this protein is the most exposed viral protein at the capsid surface and the main inducer of neutralizing antibody. As this gene was identified as a region with a high frequency of mutations in our previous analysis, we assessed by classical sequencing the frequency of point mutations in natural HRV hosts by comparing the VP1 consensus sequences at day 5 post-HRV-39 infection versus the initial sequence present in the HRV-39 inoculum for all 15 subjects. Again, the experiments were repeated in duplicate to exclude any mutation introduced by RT-PCR or sequencing. The sequence comparison revealed the occurrence of a single point mutation in three of the 15 subjects, one synonymous at HRV-39 full-length position 2711 for two subjects (P1105 and P1073) and one non-synonymous/non-conservative at position 3162 for one subject (P1062) (Table 1). In addition, a mixed population was found at position 2647. Thus, we can estimate that the VP1 mutation rate at day 5 of HRV-39 infection in human subjects is of approximately 3.1×10^−4^ mutations/nucleotides (total of 4 mutations/[15 subjects ×854 nucleotides analyzed]).

**Table 1:** Analysis of VP1 consensus sequences.

Mutations	Nucleotide	HeLa-OH					Subjects			Amino Acid
(FL position)	in Inoculum	A	B	C	D	E	P1062	P1073	P1105	
2400	C		***C∶T*** (50∶50)							***VP124 H→Y***
2464	T				***C***					***VP145 I→T***
2647	A							***A∶T*** (50∶50)		***VP1106 Q→L***
2711	A	T	T	T	T	T		T	T	VP1127 (P)
2821	G	**C**								**VP1164 G→A**
3162	T						***C***			***VP1278 S→P***

Majority mutations present in VP1 after 5 days of HRV-39 infection in five independent HeLa-OH cell experiments (A to E) and in 15 inoculated human volunteers are represented. Non-synonymous mutations are in bold and non-conservative in bold and italic. The nucleotide and amino acid positions are indicated relative to the HRV-39 reference sequence (Genbank accession # AY751783), complete genome and VP1 protein, respectively.

In comparison, eight VP1 mutations were identified in the five HeLa-OH cell experiments after 5 days of infection. The synonymous mutation at position 2711, previously observed in vivo for two subjects, was present in all HeLa-OH cells. Each of the three remaining non- synonymous mutations (the one at HRV-39 full-length position 2400 representing a mixed population) was present in only one of the five cell experiments. Based on these data, we estimate that the occurrence of VP1 mutation after 5 days of HeLa-OH cell infection with HRV-39 is of 1.9×10^−3^ mutations/nucleotides (total of 8 mutations/[5 HeLa-OH assays ×854 nucleotides analyzed]). Regarding the complete ORF, VP1 mutation frequency is higher in vitro under our conditions than in vivo in experimentally infected individuals with a reference strain. Of note, none of the mutations observed both in vivo and in vitro is located in neutralizing immunogens or within the VP1 drug-binding pocket. No mutations were found in the cis-acting replication element, which is located in the 2A gene for human rhinovirus A [22].

## Discussion

The study of HRV genome evolution is limited by the lack of animal models and the short duration of most human infections. To bypass this limitation, we analyzed the HRV genome evolution in experimentally infected human volunteers and compared our results with those obtained in an in vitro culture model. Our analysis based on 15 HRV-39 inoculated subjects first confirms that the peak viral load is reached at days 2 or 3 post-infection, as shown previously in HRV-39- or HRV-16-inoculated subjects. This correlates also with the kinetic of common cold symptoms [14], [23], [24]. Relatively high levels of viral RNA are still found 5 days' post-infection, although substantial inter-individual variations were observed and suggest that factors including the host innate immune responses (all individuals presented a serum neutralizing antibody titre of 1∶4 or less against the reference HRV-39 strain suggesting a minor effect of the adaptive-immune responses) could modulate viral replication. Most importantly, our analysis was conducted during a period of time that corresponds to the main period of transmissibility [25] and during a high rate of replication cycles.

By using ultra-deep sequencing technology, we were able to pinpoint HRV evolution at the level of a quasispecies population both in vivo and in vitro. Our data illustrate the ability of rhinoviruses to produce several new variants as rapidly as 5 days' post-infection. This represents a minimal estimate of replicating minority variants since mutations present at frequencies lower than the ultra-deep sequencing limit of detection are not represented here.

The transmission of viral populations between hosts and the subsequent mucosal infection could lead to a bottleneck effect that selects for a limited number of variants. Indeed, for other RNA viruses, such as HIV, infection might result from the selection of very few clones and it seems likely that as few as 1 to 5 viral particles could initiate an infection [26]. This effect was not observed for HRV since our ultra-deep sequencing analysis shows that most minority mutations present in the initial HRV-39 inoculum were transmitted and replicated in both human subjects and HeLa-OH cells (Fig. 2). Thus, based on our experimental conditions, it appears that HRV inter-host transmission is not associated with a strong bottleneck effect. We observed simultaneous co-infection with several minority variants together with the dominant population.

To the best of our knowledge, our study allowed to define for the first time a virus mutational map on the entire ORF in vivo. Both classical and ultra-deep sequencing methods revealed the presence of mutation hot spots along the entire coding sequence in the viral capsid genes VP1, VP2 and VP3, whereas cold spots were found in VP4, 2A, 2B, 3A, 3B and 3D. Interestingly, this is consistent with previous published data that compared the RNA genome sequence of prototype HRVs serotypes (Fig. 4B) [9]. A previous analysis based on the study of 35 HRV full-length sequences has already demonstrated that the HRV genome was under purifying selective pressure with islands of diversifying pressures located in VP1, VP2, VP3, 3C and 3D genes [27]. Our results obtained after 5 days of viral replication in human subjects and HeLa-OH cells confirm that VP1, VP2, VP3 and 3C, but also 2C genes, appeared to be under a diversifying pressure compared to others parts of the ORF (Fig. 2, 3, 4). A similar evolutionary pattern was observed in experimentally infected humans as well as in vitro, thus suggesting an evolutionary pattern common to the HRV species. Whether the presence of these mutation hot spots are linked to host immune pressure and/or to intrinsic replication constraints of the virus remains to be elucidated. However, knowledge of hot and cold spots may contribute to the identification of stable targets for new antiviral and vaccine therapies. This also contributes to our understanding of the diversity of one of the most frequent agents infecting humans.

When analyzing the occurrence of mutations in VP1 gene in both inoculated human subjects and HeLa-OH cells, we found a lower mutation frequency in vivo in humans (3.1×10^−4^) than in vitro in HeLa-OH cells (1.9×10^−3^). The mutation frequency estimations based on the ORF sequences strengthened these results (3.4×10^−4^ and 8.4×10^−4^ in humans and HeLa-OH cells, respectively) and are consistent with published data that estimate an error rate of picornavirus RNA polymerases ranging between 10^−3^ and 10^−4^ errors/nucleotide/cycle of replication [4], [5]. HRV is one of the most frequent viral agents in humans with more than 100 serotypes co-circulating that have emerged through repeated infections of short duration. The mutation frequency observed in our study is rather limited and at the lower end of what might have been expected, thus suggesting that HRV serotypes (at least for HRV-39) have already evolved over a prolonged period of time. Furthermore, in the absence of any knowledge of the number of replication cycles, we should be careful to draw any definitive conclusion between in vitro and in vivo conditions. Still, these differences could be explained by the fact that the HRV-39 inoculum might be more human-adapted as it was previously cultured only twice in WI-38 diploid fibroblasts. Second, human volunteers and HeLa-OH cells were not inoculated with the same TCID_50_/mL (10^3^ and 10^2^ TCID_50_/mL, respectively) as HeLa-OH cells did not sustain 5 days of infection as with the one used to infect human subjects. Third, the relative percentage of infected cells is likely to be higher in vitro.

The Sanger sequencing analysis performed on the entire ORF of 5 HeLa-OH and 5 NW samples showed a similar ratio of synonymous and non-synonymous mutations mainly located in hot spot regions identified by the relative mutation density analysis. Both in HeLa-OH and in human subjects, half of the non-synonymous mutations were non-conservative and were all located in VP1 and 2C genes. Interestingly, the change observed at position 2711 seems to have a beneficial effect at the genome level (synonymous) in HeLa-OH cells since this mutation emerged in all five independent experiments in HeLa-OH. This was observed only twice among the 15 human cases. Only functional studies could determine whether this specific mutation could provide a phenotypic advantage in vitro. Our data demonstrate the intrinsic ability for an already human-adapted HRV to generate new mutations in the VP1 region during the course of an acute infection in vivo. This latter point is of importance as VP1 contains sites for both antigen recognition and antiviral drug targets, such as pleconaril [6], [7]. Interestingly, after 5 days of infection, no mutations arose within the VP1 drug-binding pocket both in vivo and in vitro, but in the absence of any drug pressure.

Importantly, we did not find any mutations by both ultra-deep or classical Sanger sequencing methods within any previously known HRV functional domains (that are expected to tolerate a minimal number of mutations) and these were largely located in viral structural genes. This suggests that ultra-deep sequencing might represent a powerful tool to identify previously unknown functional domains that should be evidenced as cold spot regions. In addition, this approach is extremely useful for the study of any viral populations and quasispecies that cannot be grown in cells. Finally, this approach performed on different HRV serotypes could bring information on their respective evolutionary status as viruses having frequently circulated in humans should be more adapted and thus less susceptible to variations during the course of a human infection.

In summary, we took advantage of samples from experimentally inoculated volunteers to characterize HRV genome evolution over a 5-day course of infection, which represents the maximal window period of transmissibility. Ultra-deep sequencing analysis on minority mutation frequency and distribution allowed to identify hot spot and cold spot regions along HRV-39 ORF present both in vivo and in vitro. Our experiments suggest that HRV inter-host transmission is not associated with a strong bottleneck effect. Continued efforts to improve our understanding of HRV evolution in its natural host are essential as they represent the basis for the design of future potential vaccines and antiviral drugs.

## Materials and Methods

### Ethics statement

Written informed consent was obtained from all individuals prior to study participation. The study was approved by the Institutional Review Boards (IRB) of the University of Virginia, Charlottesville, Virginia, and the Medical University of South Caroline, Charleston, South Carolina.

### Study participants

Subjects were enrolled in a clinical study after informed consent and following review of the IRB at the University of Virginia, Charlottesville. The study aimed to assess the effect of an oral antiviral compound and only placebo-treated subjects were considered for the present study. Individuals were required to be previously healthy, between 18 to 65 years of age, and to present a serum neutralizing antibody titre of 1∶4 or less against the reference HRV-39 strain. Exclusion criteria were a history of allergic disease or nonallergic rhinitis, abnormal nasal anatomy or mucosa, or a clinically diagnosed respiratory tract infection in the previous two weeks. Pregnant or lactating women or women not taking medically approved birth control were also excluded.

### HRV-39 inoculum

The HRV-39 strain, commonly used for human inoculation studies, was initially recovered from a volunteer and cultured twice in WI-38 diploid fibroblast cultures according to standard recommendations to obtain a sufficient stock. The inoculum was tested to be safe for human in vivo usage [28].

We analyzed nasopharyngeal specimens of 19 consecutive subjects inoculated with viral stocks of ∼1000 50% tissue culture infective dose (TCID_50_)/mL administered as drops in two inocula of 250 µl per nostril given approximately 15 minutes apart while subjects were supine. NW samples were collected daily for 5 days by instillation of 5 mL of 0.9% saline into each nostril and stored at −80°C for subsequent assay.

### Quantitative real-time RT-PCR

HRV-39 RNA was TRIzol-extracted (Invitrogen, Carlsbad, CA, USA) from 190 µl of NW samples collected daily or HeLa-OH infected supernatant according to the manufacturer's instructions. As an internal control, 10 µl of standardized Canine Distemper Virus (CDV) of known concentration were added to each sample before extraction. Extracted RNA was used as a template for the synthesis of cDNA with random hexamers (Roche, Indianapolis, IN, USA) or oligo-dT primers (Roche) at 42°C using the reverse transcriptase (RT) SuperScript II (Invitrogen) according to manufacturer's instructions. cDNA was then amplified and detected in a TaqMan real-time PCR reaction using a validated human picornavirus combination of primers and probes named “Panenterhino” [29] and the CDV assay (primers: CDV-fwd: 5′-gctacccaagaaaccgtcattg-3′, CDV-rev: 5′-gcatggcagggacgagtt-3′, probe: CDV-probe: 5′-VIC-cgttcagggagtccaggactacgtcaac-TAMRA-3′), respectively, under the following cycling conditions: 50°C for 2 min; 95°C for 10 min; 55 cycles of 95°C for 15 s and 60°C for 1 min in a 7500 Applied Biosystems thermocycler. Results were analyzed using the SDS version 1.4 program (Applied Biosystems, Foster City, CA, USA). Quantitative assays were run using a 10-fold dilution series of a titrated HRV-39 stock (ATCC) that was used as a reference quantitative curve for each run. In addition, we obtained Ct values for the CDV assay in each run to control for intra- and inter-assay variability.

### Cell culture and infection

HeLa-OH cells were grown in Eagle's Minimum Essential Medium (EMEM; Lonza, Wokingham, UK) supplemented with 2 mM L-glutamine, 1 µg/mL amphotericin, 100 µg/mL gentamicin, 20 µg/mL vancomycin, and 10% fetal calf serum (FCS) at 37°C in a 5% CO_2_-containing atmosphere. The original HRV-39 inoculum (10^6^ TCID_50_/mL) used to inoculate the human subjects was diluted 10^4^-fold in 1 mL (10^2^ TCID_50_/mL) of McCoy's 5A Medium–2% FCS to infect, with this 1 mL, 80% confluent HeLa-OH in 33 mm wells for 2 h at 33°C. Cells were washed twice with PBS (Ca^2+^ and Mg^2+^ free). Finally, 1 mL of fresh McCoy's 5A Medium–2% FCS was added in each well and cells were incubated at 33°C. Supernatants were recovered and extracted 5 days' post-infection for real-time PCR analysis and sequencing. This procedure was repeated in five independent experiments.

### VP1 gene and full-length genome amplification by PCR

VP1 amplicon was obtained by amplification of two overlapping PCR fragments (primers VP1_2195_/VP1_2860_ and VP1_2626_/VP1_3325_, Figure S1). The HRV-39 ORF sequence (nucleotide 615 to 7058) was obtained by amplification of eight overlapping PCR products with primers HRV39-1 to 8 forward/reverse (Figure S1). For ultra-deep sequencing, RT was performed in duplicate and each PCR in quadruplicate to discard any mutations introduced by polymerase errors. We used Pfx50 DNA polymerase for PCRs according to the manufacturer's instructions. All amplicons were purified with the microcon columns (Millipore, Zug, Switzerland) before sequencing. PCR quadruplicates originating from the same RT reaction were pooled at equimolar concentrations and used for ultra-deep sequencing.

Both VP1 and ORF classical sequencing were performed in duplicate directly on PCR products with the same primers used for each individual PCR. Sequencing was performed with ABI Prism 3130XL DNA Sequencer (Applied Biosystems). Chromatograms were imported for proofreading with the vector NTI Advance 10 program (Invitrogen). Overlapping fragments were assembled with the contigExpress module of the vector NTI Advance 10.

### Ultra-deep sequencing analysis

#### Samples

Libraries were prepared according to the manufacturer's protocol (Illumina, Inc., San Diego, USA) using bar-coded adapters designed by Fasteris. Each library consists of eight PCR products pooled at equimolar concentration and fragmented by nebulization. After end repair to generate blunt ends and the addition of one A at the 3'ends, fragments were ligated with a modified genomic adapter containing a four-base bar-code at its 3'end and purified on agarose gel to recover fragments of approximately 300 bp. PCR amplification was performed for 15 cycles using the Phusion polymerase. Libraries were purified and quality controlled by cloning a 1 µl aliquot into a pCR4Blunt-TOPO plasmid (Invitrogen) and capillary sequencing of eight clones to verify correct constructs and inserts. We quantified the libraries using BioAnalyzer (Agilent, USA) and Q-bit (Invitrogen) and diluted to 10 nM.

#### Genome analyzer run

Libraries were pooled and sequenced on an Illumina Genome Analyzer GAII single-read channel for 76 cycles using a version 3 sequencing kit. We performed base-calling using Illumina GAPipeline-1.3.2, which produced over four million pass filter reads or 329 Mb.

#### Bioinformatics data analyses

An average of 3.5×10^5^ HRV-39 mapped reads was obtained for each sample analyzed with a mean coverage of 4100 readings per nucleotide. Short reads were first mapped with MAQ software version 0.7.1 accepting a maximum of two mismatches within the first 24 bases on the HRV-39 reference sequence (Genbank accession AY751783). Reads mapping on repeated regions were attributed randomly to one of the possible locations. The MAQ consensus sequences, including SNP detection, were generated for all regions with a minimum coverage of three bases. We conducted a statistical analysis of the counts of the number of mapped A/C/G/T to extract potential SNP positions for each position on the reference sequence. Counts are used to determine the 95% confidence interval of the probability of observing A/C/G/T at the position while assuming the probabilities to follow a beta distribution. When the confidence interval of two of the bases probability is above a 5% threshold, the position is considered as a statistically significant SNP. De novo assembling was performed using Velvet version 0.7.31 and the resulting contigs were compared using dnadiff (MUMmer version 3.20) with the MAQ consensus sequence to validate minority mutations and discover insertions-deletions (indels). All reads were also mapped with DNASTAR NGen on the HRV-39 reference sequence and on capillary sequences obtained in our laboratories. We performed visualization and minority mutation detection using DNASTAR SeqMan version 8.0.

#### Venn diagram

Significant mutations showing common loci in the four different samples and their individual overlaps were visualized in a Venn diagram using the statistical package R [30] and the R script overLapper.R (http://faculty.ucr.edu/~tgirke/Documents/R_BioCond/My_R_Scripts/overLapper.R; accessed February 1, 2010).

#### Mutation analysis along the whole HRV-39 ORF

The variation in relative frequency of a given base at a specified position is expressed as “final relative frequency–initial relative frequency” for that base at that position. The relative frequency of a given base at a given position is expressed as the base's count divided by the count of bases at that position. The algebraic sum of all variations at a given position is always zero. Density of mutations, including those which did not exhibit change in base frequencies, was represented with a Gaussian kernel density function using a smoothing band width of 0.1 kernel standard deviation. The curves are shown at the same scale and normalized so that a value of 1 is the highest density found over all genomes. Graphs, including kernel estimates, were produced with the R statistical package.

#### HRV ORF conservation plot

We conducted an RNA sequence identify plot for the ORF of an alignment covering all known human rhinoviruses (pmid:19213880). The plot was drafted using R [30] and the packages bio3d [31] and zoo [32]. All-against-all identities were assigned for each column and average; sliding windows of 30 nucleotides with a step size of one nucleotide were used to compute a moving mean sequence identity percentage that was then plotted.

## Supporting Information

Figure S1 Primers used to amplify and sequence the human rhinovirus 39 capsid protein VP1 and the entire open reading frame.
(8.94 MB TIF)
