# Supplementary figures and images for: Rhinovirus Genome Evolution during Experimental Human Infection
Source: PLoS One. 2010 May 11;5(5):e10588. doi: 10.1371/journal.pone.0010588 (PMC2868056; doi:10.1371/journal.pone.0010588)

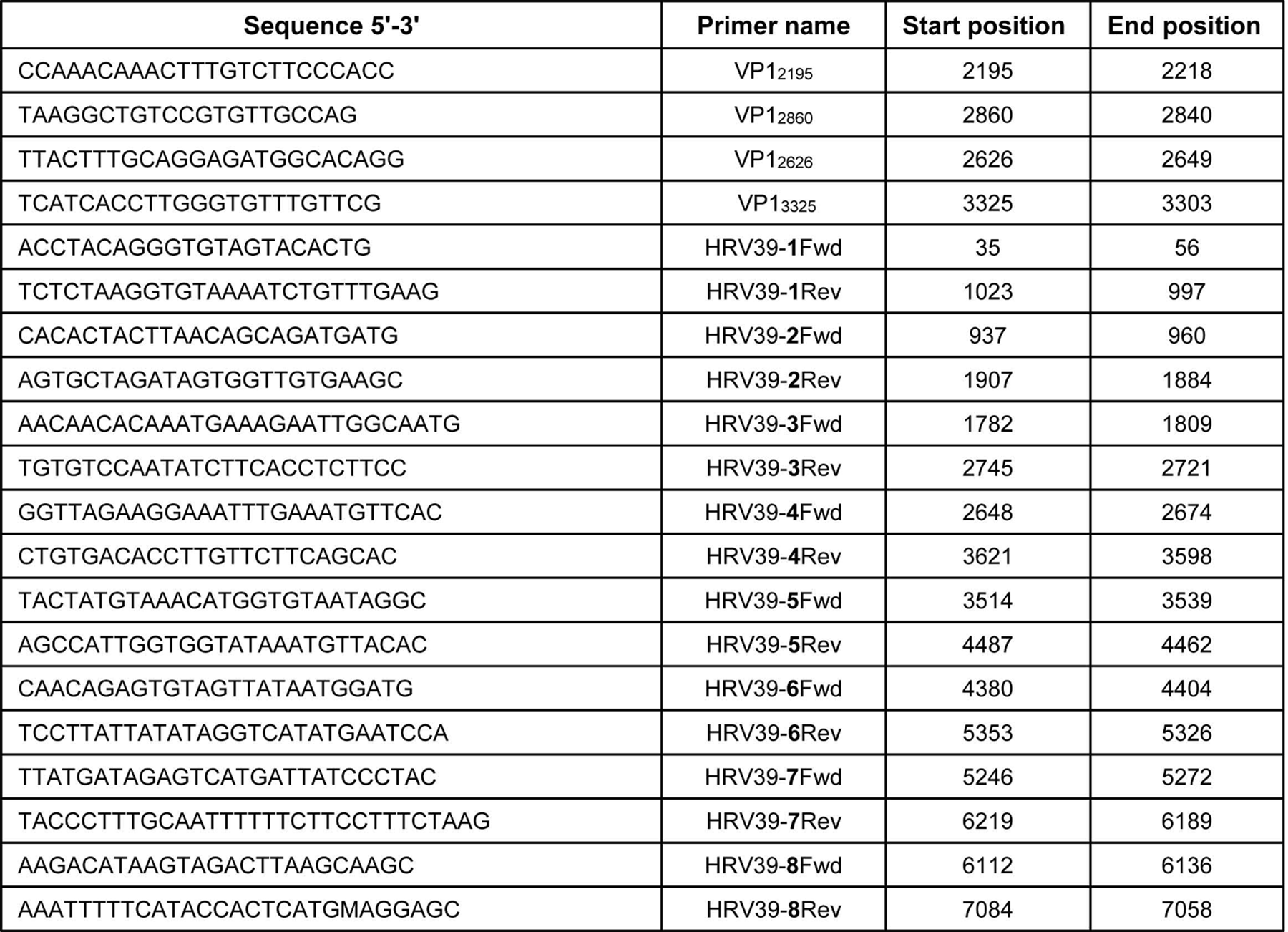

Supplement: Figure S1 — Primers used to amplify and sequence the human rhinovirus 39 capsid protein VP1 and the entire open reading frame. (8.94 MB TIF) [file pone.0010588.s001.tif]
